# Queen's Jubilee Hospital

**Published:** 1905-05-20

**Authors:** 


### QUEEN'S JUBILEE HOSPITAL.

### REPLY OF THE BOARD TO KING EDWARD'S HOSPITAL FUND.

The following statement has been circulated in reply to the report of the representatives of the King's Fund published in The Hospital on April 22nd, 1905, page 77:—

The intervention of King Edward's Hospital Fund was invited by the Board of Management in the interests of the hospital and its subscribers, owing to the trouble which arose through the unauthorised action of one of the medical staff (Dr. Morrison), who took upon himself to write a letter to her Highness Princess Louise Augusta of Schleswig-Holstein, controverting statements made to her Highness by the Board of Management relative to the new Extension Buildings and its accommodation, which letter induced her Highness to withdraw her promise to lay the foundation-stone of the new buildings.

The Board disapproved of this action on the part of the medical gentleman in question, and by resolution censured and suspended him, and subsequently he and the whole of the medical staff, with two exceptions, handed in their resignations.

The Board of Management, being thus placed in a very difficult position, immediately advertised for a new medical staff in the usual papers, and, in order that the facts might be fully made known to the Governors, and that the confidence of the public in the administration of the hospital might be continued, the Board invited King Edward's Hospital Fund to hold an inquiry into the circumstances. The Board regret that, owing to illness, the chairman was unable to be present at the inquiry.

To the great surprise of the Board, the Commissioners have made a report, accompanied by recommendations which, in the opinion of the Board, are so obviously detrimental to the best interests of the hospital, that at a meeting held on April 26th, after giving careful consideration to the report and its recommendations, the Board passed the following resolution:—

> That this Board disagree with the recommendation of the King's Fund Commissioners, and that a sub-committee be appointed to draw up a report for submission to the Board of Management.

*Clause* 8.—The fact that the Commissioners, in Clause 8, do not in any way condemn the action of the new medical staff in accepting their appointments makes it difficult to understand the suggestion of the Commissioners that the staff be asked to resign.

*Clause* 9.—The Board, without wishing to put itself in conflict with the Commissioners as to whether or not the inquiry should have been held prior to the appointment of successors to the late medical staff (Clause 9), submit that, owing to the finality of the resignations and to the expressed desire of the late staff to be relieved of their duties as early as possible, the Board had no option but to proceed forthwith to make provision for the requirements of the hospital.

*Clause* 11.—The reference in Clause 11 to the management of the hospital is founded upon conditions of the institution which for some months have ceased to exist, as was demonstrated to the Commissioners by their various references to the Minute-books, and the Board note with pleasure the opinion of the Commissioners expressed in Clause 18 that they found the hospital "in a satisfactory condition." The Board cannot agree with the view of the Commissioners that Dr. Morrison was not to blame for writing the letter to the Princess while he was still a member of the Board, and while arrangements for the laying of the foundation-stone were proceeding.

*Clause* 12.—The Board much regret to observe that their labours on behalf of the hospital are unduly depreciated by the Commissioners, and that the status of the members of the Board is unfairly criticised, and also that the statements of Mr. Tom Green, which applied to an earlier period of the hospital, and were not intended to reflect on the present management, have been made use of in a spirit other than that in which Mr. Green intended.

*Clause* 14.—The Board note with regret that the Commissioners have omitted to state that the first reason given by the late medical staff for resigning was refuted by evidence at the inquiry, no complaint, either verbally or in writing, having been made to the Board by the late staff in regard to the nursing. The nursing arrangements were under the control of the late matron, who showed her unsuitability for her position (see Clauses 14, 15, and 16) by engaging nurses without inquiry into their qualifications, by her erratic treatment of her staff, and by the condition the hospital was proved to be in when she vacated her post. There is no record of any report of insubordination on the part of nurses having been made to the Board by the late matron.

*Clause* 19.—The Board disagree entirely with the views of the Commissioners expressed in Clause 19, and are fully of opinion that a larger hospital is required in the locality in order to provide proper accommodation to deal with the pressing needs of the thousands of poor people who reside in the surrounding neighbourhoods. On this point it is interesting to note the opinion of Sir Savile Crossley, contained in the following letter written by him to the Board about 16 months ago when forwarding a cheque for £200 for the Building Fund, in which Sir Savile states that his Committee consider that a new hospital is urgently required in this district.

*Copy of Sir Savile Crossley's Letter.*

King Edward's Hospital Fund for London, 81 Cheapside, E.C., December 22nd, 1903.

The Secretary, Queen's Jubilee Hospital, Richmond Road, Earl's Court, S.W.

Sir,—I am directed by his Royal Highness, the President, to enclose cheque for £200. This sum is a special donation for this year to be applied to the Building Fund. I am also desired to inform you that my Committee consider a new hospital is urgently required in your district. Kindly acknowledge the receipt of the above.—Yours faithfully,

(Signed) Savile Crossley.

It is also interesting to note that Mr. J. Danvers Power (one of the Commissioners), in a letter which he wrote to the hospital so recently as December 1st last, expressed himself glad that building operations were in progress at the hospital, and in a further letter written by him, and dated December 21st, 1904, he forwarded a cheque for £200 for the Building Fund.

*Copies of Mr. J. Danvers Power's Letters.*

81 Cheapside, E.C., December 1st, 1904.

Miss H. S. Lane, Secretary, Queen's Jubilee Hospital, Earl's Court, S.W.

Madam,—I beg to acknowledge receipt of your letter of yesterday. We are glad to hear that building operations are now in progress at your hospital, and I will take care that this is reported to the Executive Committee at their next meeting.—Yours faithfully,

(Signed) J. Danvers Power, Honorary Secretary.

81 Cheapside, E.C., December 21st, 1905.

To the Secretary, Queen's Jubilee Hospital.

Sir,—I am directed by his Royal Highness the President to enclose a cheque for £200. This sum is sent for the purpose indicated at foot. Yours faithfully,

(Signed) J. Danvers Power.

Special donation—£200 to Building Fund.

The Board also have in their possession several letters written within the last three years by Sir Edmund Hay Currie, on behalf of the Hospital Sunday Fund, urging the Board to further extend the wards for in-patients, and in one letter the Hospital Sunday Fund threatens to withhold subscriptions from the Queen's Jubilee Hospital until "the existing arrangements for the in-patients are much improved." (See copy of letter hereunder):—

Metropolitan Hospital Sunday Fund,
Mansion House, E.C., October 6th, 1902.

Dear Madam,—I am directed by the Distribution Committee of the Fund to inform you that it will be unable to recommend any award in future to your hospital until the Committee is satisfied that the existing arrangements for the in-patients are much improved. Yours faithfully,

Edmund Hay Currie, Secretary.

The Secretary, Queen's Jubilee Hospital,
Earl's Court, S.W.

It will be seen from the foregoing letters that while the Board have been strongly urged to extend the hospital, and have received donations for the Building Fund, now that they have incurred liability by entering into a contract for building, and have actually erected new premises, they are told by the Executive of the King's Fund that a large hospital is not needed. This inconsistency on the part of the King's Fund speaks for itself.

The Board of Management are quite ready and willing to accept any recommendation that would in their opinion, or in the opinion of the Governors, be of permanent benefit to the hospital, but the Board submit that irreparable damage would be done to the institution if the recommendations of the Commissioners were adopted as a whole. It is with deep regret that the Board find that the anticipations expressed by Mr. Bavin (*see* Clause 20) have not been realised, and that the conclusions arrived at by the Commissioners are so diametrically opposed to the future prosperity of the hospital.

*Clause* 21.—The Board are also of the opinion that if the recommendations in Clause 21 were adopted in their entirety the alteration would be opposed to the best interests of the institution. To invite or compel the present efficient medical staff to resign appears to the Board to be an extraordinary proposal when it is considered that the selections were made after public notification by advertisement of the vacancies; and, taking into consideration the admittedly high character and qualifications of the present medical staff, the Board fail to see how the King's Fund, or any other body, could make a selection better calculated to serve the interests of the hospital. In admiration of the public spirit evinced by these gentlemen in responding to the Board's appeal at a most critical time, and giving the hospital their gratuitous services, the Board feel it would be most ungrateful and un-English to invite them to resign.

The Board, many of whom have for years laboured to maintain the hospital for the poor of the district, keenly feel how difficult—nay, impossible—it would be for any substituted authority to carry on the work of the hospital with only a very limited knowledge of the requirements of the locality.

While fully appreciating the promise of the Executive of the King's Fund to provide a sum of £1,000 towards the current necessities of the hospital, the Board of Management feel that they cannot advise the Governors to adopt in their entirety the recommendations of the Commissioners, but the Board suggest, for the consideration of the Executive of the King's Fund, a modification of their recommendations on the following lines:—(1) that the hospital continue to exist as a general hospital for in- and out-patients; (2) that the constitution and bye-laws be suspended for a period of 12 months; (3) that the present honorary medical staff be retained; (4) that the present Board of Management resign; (5) that a temporary Board of Management be formed to serve for one year, and then to report to the Governors; (6) that one half of the lay members of the new temporary Board be elected by the Governors, and the other half by the Executive of the King's Fund; (7) that the chairman be nominated by the Executive of the King's Fund; (8) that the medical staff, in addition, elect representatives from their own body to serve on the Board, to assist and advise on matters relating to the medical department; (9) that a competent male secretary be appointed forthwith.

This report was approved and adopted at a meeting of the Board of Management on May 10th, 1905, and a copy was ordered to be sent to the Executive of the King's Fund, the Hospital Sunday Fund, the Hospital Saturday Fund, and to each of the Governors of the Queen's Jubilee Hospital.

Signed on behalf of the Board of Management,
G. R. EVERITT, Chairman.

Queen's Jubilee Hospital, May 10, 1905.

